# Spiritual Needs and Concerns of Infertility Patients: A Literature Review

**DOI:** 10.1007/s10943-025-02507-z

**Published:** 2025-12-11

**Authors:** Annelies Hommens - van de Steeg, Inge van Nistelrooij, Alistair Niemeijer

**Affiliations:** Department of Care Ethics and Policy, University of Humanistic Studies, Utrecht, Netherlands

**Keywords:** Spirituality, Infertility, Literature review, Reproductive medicine

## Abstract

Undergoing infertility treatment greatly impacts people in every dimension of life, including their spiritual existence. Spirituality is an intrinsic part of human existence and comprises three essential attributes and two common qualities. The three key attributes are: (I) transcendence, (II) connectedness to self, others and the world, and (III) the human search for meaning and purpose. The two qualities that distinguish spirituality are (1) the capability for change and evolvement and (2) a personal way of ‘being in the world.’ However, very little attention has been paid in research to the specific spiritual needs of infertility patients. We conducted a narrative literature review to examine what is known, with the aim of coming to a thorough understanding of the spiritual concerns of patients of fertility clinics. Based on the search criteria, 48 studies from around the world were included. We found that spiritual concerns of infertility patients occur in the midst of life as it is lived. Analyzing the literature shows how the three attributes of patients’ spirituality change profoundly, while the two qualities are revealed in the fluidity and open-ended nature of this change. We describe this change as the unraveling of the experienced unity of life. We conclude that current literature lacks a framework that looks at humanity as existing in and through relations. This would enhance the understanding of the spiritual needs of infertility patients.

## Introduction

Undergoing infertility treatment greatly impacts people in every dimension of life, including their spiritual existence. It awakens spiritual needs and questions regarding the purpose and meaning of life, because the longed-for future as parent of a biological child becomes uncertain (Romeiro et al., 2017b).

Becoming a parent is widely recognized as a significant and meaningful life event (Prinds et al., 2014). In advanced economies, ‘family and children’ consistently rank as the primary source of meaning in life (Silver et al., 2021). This longing for a child reflects the prospective parents’ broader vision of what constitutes a ‘good life.’

Approximately one in 6 people of reproductive age experience difficulties in their attempts to become pregnant (WHO, 2023). Infertility thwarts the goal of becoming a parent and as such can lead to a crisis of meaning. McCarthy (2010), who conducted retrospective research among women only, concludes that confronting infertility often triggers a life crisis that alters women’s sense of self, identity and meaning and purpose in life.

In medical literature, infertility is usually defined as ‘a disease of the male or female reproductive system defined by the failure to achieve a pregnancy after 12 months or more of regular unprotected sexual intercourse’ (WHO, 2023). Innovation in artificial reproductive techniques (ART) gave people with infertility the chance to fulfill their wish for offspring. In the Netherlands, these consist of artificial insemination (AI) with own or donor semen, in vitro fertilization (IVF) and Intracytoplasmic sperm injection (ICSI) with own or donor gametes, surrogate motherhood, and pre-implantation genetic diagnosis (PGD) (Griessler et al., 2022). Treatment duration can be long and arduous, lasting from several months to several years (Boivin et al., 2011).

Moreover, a positive outcome is far from secure. In the Netherlands, live births per ART aspiration are about 22–25 percent in 2019 (The European IVF Monitoring Consortium (EIM) for the European Society of Human Reproduction and Embryology (ESHRE) et al., 2023). Many couples deal with multiple pregnancy losses during treatment or never conceive. However, the time in treatment is constructed as a hopeful time in which one is ‘not yet pregnant’ (Allan, 2007; Greil et al., 2010) and a lack of attention for the uncertainty and ambiguity during treatment intensifies patients’ struggle to make sense of their lives (Cunningham, 2014).

For many years, there has been a growing awareness that the integration of spirituality in healthcare is beneficial to both patients and clinicians. Holistic patient care—that includes spirituality—increases quality of care and can improve health outcomes and patient satisfaction (Puchalski et al., 2014). However, very little attention has been paid to the specific spiritual needs of infertility patients, let alone to the provision of spiritual care for them.

Therefore, it is essential to come to a thorough understanding of the spiritual concerns of people undergoing ART. We do this by analyzing existing qualitative literature that studies the experiences of infertility patients and creating an overview of what is currently known about their spiritual needs and concerns. In doing so, we seek a deeper and broader meaning of the experience of undergoing fertility treatment from the perspective of spiritual needs as reported by patients. But first, we operationalize ‘spirituality’ in relation to fertility treatment.

### Spirituality

Spirituality is an intrinsic part of being human, yet difficult to define, due to its deeply personal, intangible nature and the multiple dimensions that collectively shape it (Molina, 2019; Steinhauser et al., 2017). Moreover, there are many definitions of spirituality, and no consensus has been reached so far. Critics of the concept consider it to be too broad, and therefore meaningless (Salander & Hamberg, 2014). However, the risk of conceptual stretching can be mitigated by firmly anchoring the concept of spirituality. To establish the theoretical perspective for this literature review, we examined several definitions of spirituality (Molina, 2019; Perrin, 2024; Steinhauser et al., 2017; Weathers et al., 2016) for key terms (see Table 1). We chose these from a wide range of definitions, because they stem from decades of research in palliative care (Steinhauser et al., 2017) or from research in reproductive health (Molina, 2019; midwifery). We also included a definition used in some of the retrieved studies (Weathers et al., 2016; nursing) and one that resonates with the background of the main author (Perrin, 2024; Christian theology).

**Table 1:** analysis of different definitions of spirituality (all italics by the authors)

Definition	Field of inquiry	Key terms
Spirituality is a *dynamic* and *intrinsic* aspect of humanity through which persons seek *ultimate meaning, purpose, and transcendence*, and *experience relationship* to self, family, others, community, society, nature, and the significant or sacred. Spirituality is expressed through beliefs, values, traditions, and practices. (Steinhauser et al., 2017)	Palliative care	· Dynamic
		· Intrinsic
		· Seeking ultimate meaning, purpose, transcendence
		· Experiencing relationships
A key factor in the definition of spirituality is the widespread *need for humanity to search for meaning and purpose in life* and consequently *the construction of spiritual identity.* […] The definition of spirituality better suited to this study is one that involves *a person’s beliefs, experiences and feelings about the self and important relationships*. (Molina, 2019)	Midwifery	· Search for meaning and purpose
		· Identity
		· Beliefs about self and important relationships
The first element is that spirituality is *a human spiritual nature*. The *search for meaning, values, reflection, and purpose in life*, are some of the spiritual *innate qualities* that humans possess. The second element is *the capacity for transcendence and self-transcendence*. These capacities are interlinked with *intimacy, relationships with others and ultimately, with the planet.* […] The third element is that *spirituality is a ‘lived reality’, in which attitudes, practices, rituals and behaviours describe the daily lives of people*. […] The fourth and final element is the *academic study of spirituality*.’ (Perrin, 2024)	Christian Theology	· Human nature
		· Innate qualities
		· Search for meaning, values, reflection, purpose
		· Capacity for transcendence
		· Relationships with others and the planet
		· Lived reality
		· Academic study
Spirituality is *a way of being in the world* in which a person feels *a sense of connectedness* to self, others, and/or a higher power or nature; *a sense of meaning in life*; *and transcendence beyond self, everyday living, and suffering* (Weathers et al., 2016)	Nursing	· Being in the world
		· Connectedness
		· Meaning in life
		· Transcendence

Upon reviewing these definitions, we found that all of them reveal three essential attributes and two common qualities. The three key attributes are: (I) transcendence, (II) connectedness to self, others and the world, and (III) the human search for meaning and purpose. The two qualities that distinguish spirituality are (1) the capability for change and evolvement and (2) a personal way of ‘being in the world.’

Spirituality’s relation to religion is often unclear; we identify the phenomenological quality of spirituality as its distinguishing feature. Spirituality originates in the lived experience, while religion has objective qualities (e.g. a structured organization or authoritative texts and practices). Spirituality can therefore be expressed through religion but does not necessarily need to be (Weathers et al., 2016). In controversial ethical practices like ART, religious patients will have to negotiate their religion’s views on reproduction and make peace with any conflicts between them and their lived experience of a desire for a child (Klitzman, 2018). Hence, spirituality, as we see it, transcends religious boundaries and is an intrinsic aspect of human existence, making it a vital component of healthcare (Leget, 2013).

**Fig. 1:**
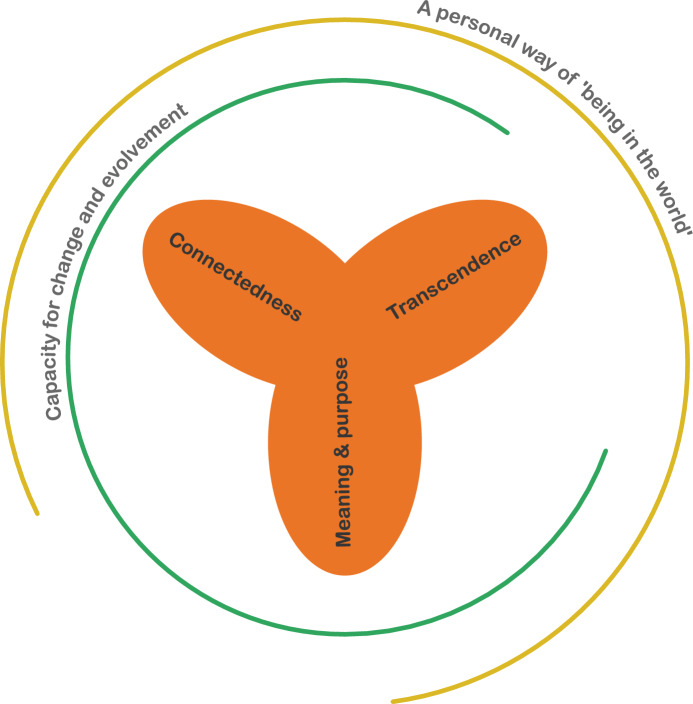
visualization of attributes and qualities of spirituality (made with Adobe Illustrator)

Further unpacking the concept, we identify the attributes of spirituality to point to the ‘what’ and the qualities to point to the ‘how’ of the concept. Transcendence, connectedness and searching for meaning and purpose are the way spirituality reveals itself in the lives of people. Spirituality forms the nexus of these three attributes. The way these attributes reveal themselves is as lived experience, in which they change and evolve (see Fig. 1).

We will now examine the attributes further, as this is critical for understanding the concept (Rodgers, 2000 in Weathers et al., 2016).

*I: Transcendence* is a multifaceted concept related to the different ways in which humans understand their lives embedded in an ultimate reality (Bayne, 2018; Stoker & Merwe, 2012). We understand transcendence through the lens of the philosophical concept of ‘social imaginaries’ (Taylor, 2007). These are defined as the ways in which people imagine their social surroundings, not in theoretical terms but carried in images, stories and legends (Alma et al., 2018; Taylor, 2007). They are shared by large groups of people (Alma et al., 2018) that live out their lives in a practice that “largely carries the understandings” mediated through these images and stories (Taylor, 2007). 

Social imaginaries are transcendent, normative, and implicit. They are driven by a deep longing for a meaningful life, rooted in bodily experiences (Taylor, 1989). Hence, they favor some ways of living above others. However, we are mostly not aware of this normative pull. Imaginaries often remain unarticulated, unless some kind of challenge makes it necessary to reflect on the normative imaginaries about who we should be and how we should act in life (Taylor, 1989, 2007). The desire to become a parent can be seen as connected to some social imaginary, as undergoing infertility treatments is worthwhile because of the transcendent meaning of this desire.

*II: Connectedness* points to the fact that we all live in relationships and connections to others. We distinguish two dimensions (cf. Ricoeur, 2005; cf. Schües, 2016). First, we are part of a certain generation that lives in a certain place, time and culture. Second, we are connected to those who came before us and to future generations, for example, through our family lineage.

Our self-understanding, as well as the desires that we commit ourselves to, like becoming a parent, are embedded in these dimensions. The meaning we attach to our desires arises out of these dimensions, because they give us a specific understanding of our desire (Taylor, 1989). Hence, in undergoing infertility treatment, patients do not only relate to their personal desire to become parents, but also to the context in which this desire arises.

*III: The human search for meaning and purpose* describes the tentative, intuitive search for our experiences to ‘make sense,’ the moment our deepest desires, wishes and ways of living are put under pressure (Taylor, 1989). Infertility as an, often unexpected, life crisis (Romeiro et al., 2017a; Roudsari et al., 2007; Schick et al., 2016; Stenström & Pargman, 2021) makes patients aware of the normative pull of their desire and raises profound questions about what life is really ‘all about’ and how we can find ways to live through and in this experience.

To conclude this operationalization of spirituality, we state how we use our analysis of the concept as a heuristic lens for reviewing the selected literature. In order to understand the spiritual needs of infertility patients, we look at the way transcendence, the human search for meaning and purpose and connectedness reveal themselves in lived experience and describe the changes and adjustments that people make in the vicissitudes of life.

## Method

As our goal is to provide an overall summary of what is known of the spiritual needs and concerns of patients of fertility clinics, we conducted a narrative literature review. This method provides the flexibility to select and analyze literature from different academic disciplines, allowing for a comprehensive examination and interpretation, as well as room for the iterative process of refining the theoretical perspective while assessing the literature (Ferrari, 2015). To minimize bias, we assessed the quality of the studies by looking at study objectives, study design, data collection and analysis, results, and limitations. These elements are recognized as indicators of quality in checklists for literature reviews and qualitative research (Page et al., 2021; Tong et al., 2007).

In line with our operationalization of spirituality as outlined above, we employed a wide range of search terms to capture the ‘what’ of the concept. A preliminary search helped refine the following query as appropriate: Existential OR spiritual OR “sense making” OR “meaning making” OR “purpose in life” OR “philosophy of life.”

We also let the three attributes and two qualities of our theoretical concept guide us in identifying data and analyzing the included studies. For this reason, we did not require the articles to have a definition of spirituality, as long as they provide in-depth information about one or more of the three attributes of spirituality, acknowledge their personal nature and their capacity to change and evolve. Moreover, in our analysis we focus on the way in which participants describe their experiences as fertility patients. Quotes from participants are deemed to be highly relevant, as they offer us insight into their lived experience.

### Search Strategy

A search using the above-mentioned query was conducted across four databases: EBSCO, PsycINFO, PubMed, and Web of Science. Since we wanted to focus on the patient’s perspective, we also included the search terms *patient OR woman OR women OR man OR men OR couple OR people.* In this way, studies that focus exclusively on the views of caregivers were excluded.

Because we aimed to find a broad range of studies on infertility, we used the synonyms *subfertility* and *subfecundity,* as well as the phrase “fertility issues” as search terms. We evaluated other reviews on this topic and found that our search terms are in line with these. We did not include the search terms “childless not by choice” or “unwanted childlessness” because these terms indicate that achieving parenthood is no longer possible. Undergoing fertility treatments is characterized by a profound uncertainty whether one will become a parent (Greil et al., 2010), causing its own specific spiritual questions. When treatment is concluded, uncertainty and ambiguity no longer characterize the situation, which leads to different spiritual needs (e.g., coming to terms with the fact that one will not become a parent).

### Inclusion Criteria

Fertility issues may cause people to seek treatment that is offered in hospitals and private clinics around the world. They long to make the transition from ‘trying for baby’ to ‘expectant parent’ and the medical technologies offered by the clinic provide the hope and expectation that this will happen (Allan, 2007). The time as a patient is characterized by undergoing diagnostical and treatment interventions and is terminated by either a viable pregnancy or the decision to stop after one or more unsuccessful treatments (Peddie et al., 2005; Romeiro et al., 2017b). Being an infertility patient can be seen as a state of ‘being in-between’ (Allan, 2007) and the tension of this liminality can provoke spiritual concerns that might be different from those of people who have not yet started or already ended treatment (Romeiro et al., 2017a).

For this reason, we included only those studies as a primary source that focus on the views of patients of infertility clinics. However, we did not distinguish between types or stages of treatment, nor pre-exclude any care-setting (hospital, focus clinic) or geographic location; provided fertility treatment is an available option. Excluded were retrospective studies, in which the participants look back on their experiences as patients in a fertility clinic.

Because we view spirituality as a dynamic and evolving lived experience, we included studies that focus on how spiritual needs emerge during treatment. This is also the reason we do not include any prospective studies; the views of people who are not finding themselves amidst the experience of fertility treatment could be not only speculative but also theoretical, different from the actual experience. Lastly, we included articles where part of the respondents had already finished treatment as a secondary source. Their inclusion served the purpose of capturing the difference in needs (if any) of current and former patients.

Sexual orientation and marital status also function as major barriers to fertility care. In many countries, treatment is only available to heterosexual, married couples (Fertility Europe and European Parliamentary Forum for Sexual and Reproductive Rights (EPF), 2024). In some countries, including the Netherlands, this is not the case (EPF 2024; Griessler et al., 2022). We included studies with patients of any age, race, gender, sexual orientation, or marital status. Studies of patients with other co-morbidities (for example, infertility due to cancer) were also included, as we believe these people confront similar spiritual questions concerning their hoped-for parenthood.

Spirituality concerns lived experiences and changes and evolves over time (Taylor, 1989, 2007). Our research aims to gain insight into contemporary spiritual needs. Because of these reasons we set the period for publication of studies from 2004 to 2024. Articles were included when they were written in English. Further, we included only review articles, qualitative and mixed-method studies from peer reviewed journals, since data from purely quantitative studies did not fit our qualitative outlook of exploring experiences and meanings (Greil et al., 2010). All articles included are available in full text via either open source or the libraries of Utrecht University, the National Library of the Netherlands or the *Staatsbibliothek zu Berlin*.

The screening of the articles was done by the first author, using Rayyan, an online tool for literature reviews. This resulted in the removal of duplicates and a list of included and excluded articles, with the reasons for their in- or exclusion. This list was then reviewed by the second and third authors, and differences of estimation were discussed until a consensus was reached. In addition, key articles were identified and snowballed for further suitable references. These references were screened for inclusion as well. All articles included were summarized for further assessment purposes.

### Analysis of Included Articles

All included articles were read in full by the first author, who described the major characteristics, and any spiritual themes as emerged from the study. The articles were then uploaded to Atlas.ti, a program for qualitative analysis, where a further thematic analysis was made by the first author, listing all that was mentioned about spiritual needs. An iterative process identified and named twelve overarching themes. These themes were then discussed among the authors and further clustered under the three attributes of spirituality that form part of our heuristic lens. This second analysis was shared and discussed with fellow researchers. Their feedback was used to further shape the results and the way they are presented.

## Results

**Fig. 2:**
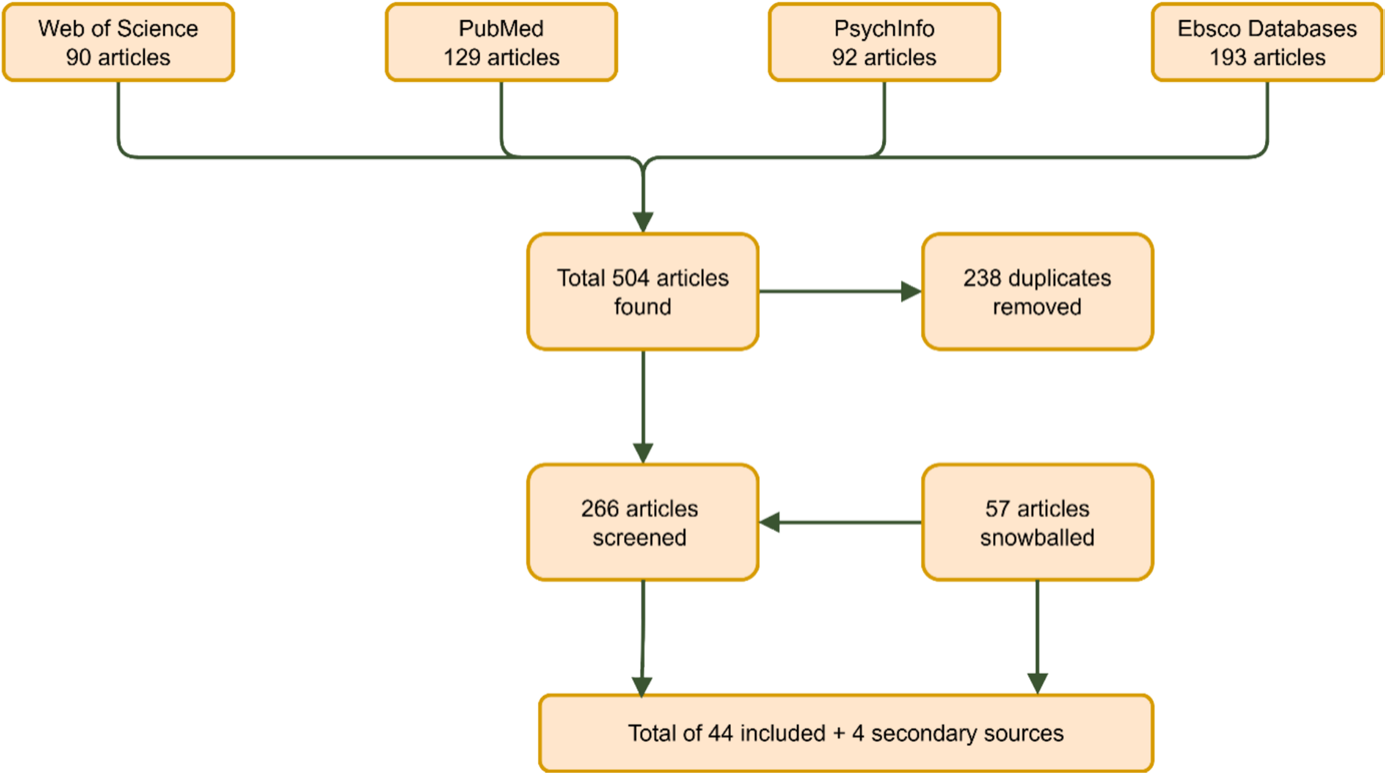
Flowchart of literature search (made with draw.io)

Based on the search terms used, 504 articles were found, which left 266 articles after removing duplicates. Snowballing of 5 articles identified as key articles added another 57 articles. In total, 48 articles met the inclusion criteria and quality standards, of which 44 were a primary source and 4 a secondary source (Fig. 2). All articles were identified with a number (see Appendix 1).

Medical peer-reviewed journals (n = 22) were the main source, followed by nursing (n = 11), media and communication studies (n = 5), psychology (n = 4), religion (n = 4) and sociology (n = 1). One publication was published in a regional journal (47).

Most participants of the studies were women (n = 28) or couples (n = 10). A few studies focus on the experiences of men exclusively (n = 4). One study had both male and female respondents, but it is not clear if this concerns couples (Klitzman, 2018). None of the studies report other than cisgendered patients in a relationship. The included studies were set in Botswana, China, Canada, Germany, Ghana, India, Iran, Italy, Jordan, Mali, Morocco, Portugal, South-Africa, Spain, Sweden, Taiwan, Turkey, the UK and the USA. In order to understand the experience of patients of infertility clinics, the most used designs were phenomenological (31%) and grounded theory (13%). Most studies did not specify the stage of treatment that patients were in, with the exception of studies that focused on Ovarian Hyperstimulation Syndrome (9), hysterosalpingography (15), spontaneous pregnancy loss after treatment (18), unsuccessful treatment (26, 33) and the time between embryo transfer and pregnancy test (43).

### Presentation of Themes

Our analysis of the studies demonstrates that spiritual concerns of infertility patients occur in the midst of life as it is lived with and among others and cannot be confined to the inner life of the patient. We first present an overview of the main themes with quotations from the original studies and references (see Appendix 2).

We subsequently relate the themes to different attributes from our operationalization of spirituality that form our heuristic lens (transcendence, connection to others and self and search for meaning and purpose), paying attention both to how these attributes reveal themselves through the cluster of themes (‘what’) and the ‘how’ of patients navigating the changes in their lives. We give examples from the studies reviewed, referring with numbers in parentheses to the original studies. Information about the studies’ location and design can be found in Appendix 1. For reasons of clarity, we discuss the connection to self and the connection to others under separate headings. We conclude by answering the question of what these changes mean for patients’ spirituality as a whole.

#### Transcendence: Desire Under Pressure

When we ask what transcendence is in relation to infertility treatment, we find that it reveals itself as an intense desire of patients to become parents. This is a deep longing that is central to one’s being (7, 14, 22, 25), often heightened by undergoing treatment (17, 25). This desire is so intense because having children represents the fullness of life or an intimate connection with someone that really belongs to you.

When we look for examples of the normativity of this desire, we find a study from Taiwan that shows how the virtue of filial piety forms a background to the expectation that couples continue the family line (9). Another study (4) shows how the difference between an imaginary of family as nuclear (in the UK) or extended (in Pakistan) could account for differences in the experience of being a fertility patient. This study infers that the impact of infertility might be greater in non-western countries, because of more pressing norms around family formation.

Transcendence reveals itself through spiritual distress; in the form of loss of purpose, loss of normal reality and the loss of the experience that time flows toward a meaningful future (‘how’). Treatment has taken away the security that life can be planned (13). However, stopping treatment can be viewed as ‘giving up hope’ (17) and therefore also no solution.

#### Connectedness to Self: Identity Shaken to the Core

A profound change takes place during treatment in the connection to self. This shows itself in the loss of familiarity with the embodied self; a questioning of identity, and loss of agency.

During treatment, men and women start to perceive their bodies as deficient (4, 11, 27, 37). Under the gaze of the clinic (12), women’s bodies (in particular) are ‘turned inside out’ (47). Women experience their embodied self as pregnant after embryo transfer, establishing an emotional and moral bond with the embryo (43). Hence, after transfer, they anxiously try to determine what body symptoms might be indicative of an ongoing pregnancy (12, 47). And even when pregnant, the view of a deficient body continues (13).

A study that highlights the normative aspect of reproductive capability (19) explains the loss of identity. Reproduction alone is capable of defining manhood and womanhood; hence one is not a real man or a true woman because of one’s inability to conceive. Lastly, patients’ loss of agency presents itself as paradoxical. They experience both helplessness and a heightened sense of responsibility (12, 41). Where it was presumed that the body knows how to procreate, it is now implied that one can and must make an effort to get pregnant (47).

#### Connectedness to Others: Out of Place

When people undergo infertility treatment, the way they belong with others is questioned. They have to relate to previously unquestioned communal imaginaries about pregnancy that have strong transcendental connotations as parenthood is understood as a higher form of being, especially for women (11, 40). In societies like Iran (5), Jordan (34) and Turkey (23, 25), infertility is seen as a ‘women’s problem,’ resulting in pressure on women to consider divorce or polygamy.

As a result of this loss of belonging, patients start looking for other communities where their stories are met with attention, empathy, and understanding. These new communities are largely found online (19, 31, 37, 46, 47). They provide a lifeline for patients and the connections made there can become so significant that they replace those with family and friends (47).

Between partners, the shared expectation that this partnership would involve children is questioned, which reveals itself in a restructuring of the relationship. A study on the attachment of a couple undergoing fertility treatment (7) describes this as a fluid, open-ended process. Hence, undergoing treatment as a couple has the potential to both existentially threaten and transform a relationship.

#### Searching for Meaning and Purpose: How to Shape Life Again as an Infertility Patient

While it is uncertain if life will unfold toward a meaningful future, many patients find themselves in a searching process. The ‘what’ of this search is described as looking for a deeper meaning that patients can now ‘live with’ (40). This reveals itself as looking for explanations of ‘what is going on’ medically (2, 13, 15), or as grappling with the uncontrollability of pregnancy, and therefore the reliability of all of life (2,13, 27).

Patients for whom religion or faith is of importance also reflect on meanings offered in their religion to see if these make sense to them (27). A few studies report on how this searching process leads to finding a new ‘essence’ (6) or sense of purpose in life, for example, in meaningful connections with children (4, 41) or a deeper understanding of the world’s suffering (6).

## Discussion

### Limitations

Acknowledging this review’s inevitable limitations is important. The first limitation concerns the lack of academic consensus regarding the concept of spirituality. Although we attempt to address this by analyzing a range of definitions, most of the studies included use the concept in a broad and often implicit way. Nevertheless, according to the authors, all of them engage—either directly or indirectly—with spirituality in relation to (in)fertility. We recognize that our review—pioneering in the field of spiritual needs of fertility patients—runs the risk of bringing together studies that might differ in their understanding of the concept.

Secondly, this review offers an overview of spiritual needs and concerns of people who are undergoing fertility treatments. Since only English and Dutch studies were included, generalizability across cultural contexts may be limited. Furthermore, we excluded both prospective and retrospective studies, focusing on the time in treatment only. As a result, this review does not offer information on how people cope with involuntary childlessness or reflect on their own spiritual journey after completing treatment. Nor does it include insights into the specific spiritual concerns of those who are unable or unwilling to undergo ART.

Thirdly, the number of included studies focusing specifically on the experiences of men or couples is limited, even though the studies reviewed suggest that spiritual needs emerge across gender and are often experienced relationally, within couples rather than individually (Glover et al., 2009; Romeiro et al., 2017a, 2017b).

Lastly, although treatment duration can last years, very few studies were found that take a longitudinal approach. Consequently, this review offers only preliminary insights into how spiritual needs and concerns may change and evolve over the course of treatment. 

### Conclusion

Analyzing the literature shows how the three attributes of patients’ spirituality change profoundly, while the two qualities are revealed in the fluidity and open-ended nature of this change. Change is revealed as a contextualized experience. Society’s social imaginaries about pregnancy and parenthood prove to be inadequate to make sense of the experience of undergoing fertility treatment. An urgency then arises to seek meaning and purpose in life. Patients’ search is undertaken in a context where others do not sense this inadequacy, resulting in the experience of self-deficiency and disconnection from others.

To describe the overall impact of this experience, two studies emphasize the chaos and disruption that threatens the whole of patients’ existence (2, 9). In the midst of this situation, they cannot but attempt to reconstruct the wholeness of life. While the three attributes of spirituality are inextricably intertwined, these observations prompt us to describe the spiritual predicament of infertility patients as an unraveling of the experienced unity of life, like a rope that frays and disintegrates.

#### Interpretations

The literature shows that undergoing fertility treatment leads to a disintegration of the experienced unity of life. Our results are in line with the literature on infertility and spirituality. Nevertheless, our review adds knowledge, because it examines the interconnectedness in the different parts of life that lead to emerging spiritual needs. The longing for a child as an intimate form of connectivity, for example, is paradoxically connected to a loss of connection with others that make up patients’ communities.

Our first point of discussion concerns the strong ties between the spiritual needs of infertility patients and social imaginaries of pregnancy, children, and parenthood. The narratives that carry these imaginaries are not only descriptive, but also normative (Lindemann, 2014). They emphasize, for example, the ‘normalcy’ of the ability to conceive, sensed by patients as a need to be(come) ‘normal.’

The disintegration of life is placed in the context of these normative imaginaries. In reviewing the literature, we find that throughout the world, a life with children is perceived as a fuller form of living than a life without children and therefore people who cannot conceive are considered incomplete. However, social imaginaries are shaped differently from country to country. The literature reasons that social imaginaries are more present, and therefore more pressurizing, in non-developed societies (see also Greil et al., 2010).

We would like to contend that, following the view of Taylor (2007), social imaginaries may be more implicit in western societies, but not necessarily less normative. The secularism and neoliberalism that characterizes these societies have a particular imaginary of, for example, personal responsibility and maximal productivity (Browne, 2022; Taylor, 2007). It is worth exploring how more secular imageries, like the one of parenthood as a consciously chosen, self-controlled ‘project’ (cf. Bueskens, 2019), provide us with the material for constructing the desirability of children.

The Dutch society is interesting in this regard, since it is characterized by a ‘fertility paradox’: Although it is a highly secular society without good government policies that support families, fertility rates are relatively high (Mills, 2015). In such a context, raising families is both a highly individualized, personal ‘choice’ and something entirely ‘normal’ and ‘self-evident.’ This paradox indicates that the motivation to start a family cannot be understood without paying attention to its contextuality. In a subsequent empirical inquiry into the lived experience of infertility patients in the Dutch context, we will shed more light on the social imaginaries that are available to infertility patients in their search for meaning and purpose.

In addition, our review shows that the spiritual unraveling that can result from undergoing fertility treatment is a complex process in a relational context. The spiritual concerns in infertility are at its core relational, rather than individual inner contemplations. From the literature, it is clear that undergoing treatment disintegrates the relationship to self and body, partner and community. Although the relational nature of reproductive medicine, especially the intimate connection between partners and between woman and embryo, is recognized in other research (Cousineau et al., 2006; Samorinha et al., 2014), we have not found research using a relational framework for conceptualizing the spiritual concerns of patients.

For a deeper understanding of the spiritual needs of infertility patients, we need a framework that looks at humanity as existing in and through relations. The academic field of inquiry of care ethics has developed such a framework to explore core needs of patients, for instance, in maternity care (Waal & Nistelrooij, 2022), care for young people with mild intellectual impairments (Nistelrooij & Niemeijer, 2023) and at the end of life (van Wijngaarden, 2016).

The studies reviewed show that belonging is of vital importance to fertility patients. Belonging means that their stories are welcomed in communities that empathize with them, making it easier, for example, to bear the insecurity that comes with inhabiting their new reality as patients. Hence, belonging fosters identity and facilitates finding meaning and purpose.

However, the process of reconfiguring identity and finding meaning through the act of telling one’s story could benefit from a deeper understanding, for example, through the lens of narrative identity (Ricœur, 1992). This can show insight into how the meaning of ‘epistemically opaque’ experiences (Lindemann, 2015), for example, an unsuccessful treatment experience, can be constructed in a way that ‘fits’ the person and is restorative of identity and purpose.

In this review, we found that existential ambiguity is one of the key characteristics of the process of undergoing fertility treatment. The studies reviewed link this to the role of the infertility clinic, patients’ struggle for control of the situation and the waves of hope and despair they deal with. Although studies show patients’ changed perception of time, examining the core experience of ambiguity from the perspective of time would be beneficial. Infertility treatments are entirely orientated to the future: It is all about a future pregnancy and child. This orientation is understandable and undeniable but also imbued with normativity. The present situation becomes subordinated to the projected future.

The concept of futurity, as described by the feminist philosopher Victoria Browne, refers to the normativity of certain future horizons (Browne, 2022). In this case, it is the social imaginary of pregnancy that conceals the reality of contingency and ambiguity that is experienced by patients. Disentangling the fundamentally ambiguous situation of undergoing treatment from the normative future-orientated perspective of pregnancy deepens understanding of patients’ spiritual struggles.

Our last takeaway concerns the lack of inclusivity in the language used to research the experiences of infertility patients. In all studies included, patients were referred to as women and men, without it being clear if they identified as such. Adherence to reproductive norms and gender roles by both respondents and researchers was found throughout the studies.

This does make clear that language concerning fertility patients still is very much exclusive and thus keeps promoting heteronormative models of pregnancy and parenthood. This is paradoxical, as experiencing infertility does mean that patients themselves do not fit those models. It is therefore vital that the experiences of fertility patients who do not identify with a hetero cis-normative identity get more attention in research.

## Data Availability

Data sharing is not applicable to this article as no new data were created or analyzed in this study.

## Appendix 1 Included literature

**Table:**

ID no	References	Year	Title	Study location	Study design	Source
1	Aksoy Derya et al	2022	Determining the Cultural Care Needs of Infertile Couples in Turkey: A Qualitative Study Guided by the Cultural Competence Model	Turkey	Phenomenology	1st
2	Allen	2006	Experiences of infertility: liminality and the role of the fertility clinic	UK	Ethnography	1st
3	Annan-Frey et al	2023	Lived experiences and coping strategies of persons seeking infertility treatment in the Kumasi metropolis: a descriptive phenomenological study	Ghana	Phenomenology	1st
4	Batool and De Visser	2016	Experiences of Infertility in British and Pakistani Women: A Cross-Cultural Qualitative Analysis	UK	Phenomenology	1st
5	Behboodi‐Moghadam et al	2013	Experiences of infertility through the lens of Iranian infertile women: A qualitative study	Iran	Content Analysis	1st
6	Boz and Okumuş	2017	The ‘‘Everything About the Existence’’ Experiences of Turkish Women With Infertility: Solicited Diaries in Qualitative Research	Turkey	Phenomenology	1st
7	Brigance and Brigance	2023	A Qualitative Study of a Couple Experiencing Reproductive Trauma: An Attachment Perspective through a Duo Ethnography	United States	thematic analysis	2nd
8	Bute	2009	“Nobody Thinks Twice About Asking:” Women with a Fertility Problem and Requests for Information	USA	communication theory	2nd
9	Chang and Mu	2008	Infertile couples’ experience of family stress while women are hospitalized for Ovarian Hyperstimulation Syndrome during infertility treatment	Taiwan	Phenomenology	1st
10	Cipolletta and Faccio	2013	Time experience during the assisted reproductive journey: a phenomenological analysis of Italian couples’ narratives	Italy	Phenomenology	1st
11	Clarke et al	2008	The Continuity and Discontinuity of the Embodied Self in Infertility	Canada	embodiment /embodied self	1st
12	Cunningham	2014	Lost in transition: Women experiencing infertility	UK	narrative/hermeneutic	1st
13	Cunningham and Cunningham	2013	Women’s experiences of infertility—towards a relational model of care	UK	narrative/hermeneutic	2nd
14	Dyer	2004	`You are a man because you have children’: experiences, reproductive health knowledge and treatment-seeking behaviour among men suffering from couple infertility in South Africa	South Africa	grounded theory	1st
15	Fernández‐Sola et al	2016	Experiences of Spanish women undergoing hysterosalpingography as part of the infertility process: a phenomenological study	Spain	Phenomenology	1st
16	Ghelich-Khani et al	2021	Psycho-social experience of oocyte recipient women: a qualitative study	Iran	content analysis	1st
17	Glover et al	2009	What does having a fertility problem mean to couples?	UK	thematic analysis	1st
18	Harris and Daniluk	2010	The experience of spontaneous pregnancy loss for infertile women who have conceived through assisted reproduction technology	Canada	Phenomenology	1st
19	Harrison	2014	Online negotiations of infertility: Knowledge production in (in)fertility blogs	UK	Haraway: situated and embodied knowledges	1st
20	Hasanbeigi et al	2017	Assessment of the problems and needs of clients referred to infertility centers: a review study	Iran	traditional review	1st
21	Hasanpoor-Azghdy et al	2014	The emotional-psychological consequences of infertility among infertile women seeking treatment: Results of a qualitative study	Iran	qualitative content analysis	1st
22	Hess et al	2018	Infertility, Psychological Distress, and Coping Strategies among Women in Mali, West Africa: A Mixed-Methods Study	Mali	mixed-method	1st
23	Höbek Akarsu and Kızılkaya Beji	2021	Spiritual and Religious Issues of Stigmatization Women with Infertility: A Qualitative Study	Turkey	phenomenology	1st
24	Jafarzadeh-Kenarsari et al	2015	Exploration of Infertile Couples’ Support Requirements: A Qualitative Study	Iran	Content Analysis	1st
25	Karaca and Unsal	2015	Psychosocial Problems and Coping Strategies among Turkish Women with Infertility	Turkey	Content Analysis	1st
26	Kissiwaa and Fouché	2024	Ghanaian women’s experiences of unsuccessful in-vitro fertilisation treatment, unravelling their meanings: a Heideggerian hermeneutic phenomenological study	Ghana	Phenomenology	1st
27	Klitzman	2018	How Infertility Patients and Providers View and Confront Religious and Spiritual Issues	US	grounded theory	1st
28	Roudsari and Allan	2011	Women’s Experiences and Preferences in Relation to Infertility Counselling: A Multifaith Dialogue	Iran	grounded theory	1st
29	Roudsari et al	2014	Iranian and English women’s use of religion and spirituality as resources for coping with infertility	Iran	grounded theory	1st
30	Loke et al	2012	Experiences of sub-fertility among Chinese couples in Hong Kong: a qualitative study	Hong Kong	Phenomenology	2nd
31	Malik and Coulson	2008	The male experience of infertility: a thematic analysis of an online infertility support group bulletin board	UK	essentialist/realist framework	1st
32	Mogobe	2008	Denying and Preserving Self: Batswana Women’s Experiences of Infertility	Botswana	denying and preserving self	1st
33	Mosalanejad et al	2014	Increasing and decreasing factors of hope in infertile women with failure in infertility treatment: A phenomenology study	Iran	Phenomenology	1st
34	Obeisat et al	2012	Adversities of being infertile: the experience of Jordanian women	Jordan	thematic analysis	1st
35	Olafsdottir et al	2012	Nordic couples’ decision-making processes during assisted reproduction treatments	Nordic countries	grounded theory	1st
36	Özkan et al	2013	A Case Study Based On Watson’s Theory of Human Caring: Being an Infertile Woman in Turkey	Turkey	Case study	1st
37	Patel et al	2019	I feel like only half a man: Online Forums as a Resource for Finding a "New Normal" for Men Experiencing Fertility Issues	UK	thematic analysis	1st
38	Refaie et al	2020	Pictorial and spatial metaphor in the drawings of a culturally diverse group of women with fertility problems	UK	Case study	2nd
39	Romeiro and Caldeira	2019	The Human Responses and Nursing Diagnoses of Those Living With Infertility: A Qualitative Systematic Review	Portugal	systematic review	1st
40	Romeiro et al	2017	The Spiritual Journey of Infertile Couples: Discussing the Opportunity for Spiritual Care	Portugal	Discussion	1st
41	Romeiro et al	2017	Spiritual aspects of living with infertility: A synthesis of qualitative studies	Portugal	traditional review	1st
42	Roudsari et al	2007	Looking at infertility through the lens of religion and spirituality: a review of the literature	UK	literature review	1st
43	Şahiner and Boz	2022	Experiences of women undergoing infertility treatment from embryo transfer until pregnancy test and their conceptualization of their embryo	Turkey	phenomenology	1st
44	Sambasivam and Jennifer	2023	Understanding the experiences of helplessness, fatigue and coping strategies among women seeking treatment for infertility—A qualitative study	India	Phenomenology	1st
45	Schick et al	2016	Exploring involuntary childlessness in men—a qualitative study assessing quality of life, role aspects and control beliefs in men’s perception of the fertility treatment process	Germany	Grounded Theory	1st
46	Stenström and Pargman	2021	Existential vulnerability and transition. Struggling with involuntary childlessness on Instagram	Sweden	Interview/Online Ethnography	1st
47	Stenström	2022	Involuntary childlessness online: Digital lifelines through blogs and Instagram	Sweden	Phenomenology/Ethnography	1st
48	Zaidouni et al	2020	What are the needs of infertile Moroccan couples in Assisted Reproductive Technology?: Exploratory qualitative study in the first fertility public center in Morocco	Morocco	Content Analysis	1st

## Appendix 2 Overview of themes with quotations and references

**Table:**

Spirituality			Citation/example	References
Transcendence	Strong desire to become parents		“To be a mother is the most important thing that any woman wishes; childlessness is the biggest problem in my life.” (34)	4, 6, 7, 10, 13, 14, 15, 17, 22, 23, 25, 32, 34, 35, 38, 40, 41
	Meaning of having children	Completeness/wholeness of life	“Sexuality is of secondary importance to us. [Once] we have a child, I don’t want anything else anymore.” (1)	1, 3, 4, 6, 10–13, 17, 24, 30, 34, 41, 45
		Relatedness	“Leaving a genetic mark is extremely important for me now. The longer down the road we’ve travelled the more compelling it has become. A child is a symbol of our love and also a record of us … […]” (Diane, LS4) (13)	1, 3, 4, 5, 9, 13, 16, 17, 30, 32, 41, 43
	Spiritual distress	Loss of meaning and purpose	M: “It is hard to get back on with life because you feel like saying well what the hell are we bothering for?” F: “You feel there’s no purpose to anything.” (17)	1, 2, 4, 6, 7, 10, 12, 17, 18, 21, 26, 27, 29, 33, 34, 36–38, 40, 41, 44
		Loss of normal reality	“Hospital is like a parallel planet but in a different time and place … especially weird and unrealistic.” (12)	1, 2, 4, 6, 10, 12, 13, 16, 17, 21, 25, 26, 35, 43- 46
		Being in limbo	“Time stops because the moment to do what one must do never arrives.” (Woman) (10)	2, 7, 10, 12, 13, 15, 17, 21, 23, 24, 26, 27, 29, 30, 34–36, 38- 41, 45, 47
Connection to self	Discontinuity of embodied self		“I am critical of my body in many ways … like it has failed me, while it was strong and healthy all my life. This one thing negates all other things about my body.” (Tina: UK, 37) (4)	1, 2, 4, 7, 10–13, 15, 19, 21, 26, 27, 34, 35, 37, 38–47
	Questioning identity		“I’m wondering what’s wrong with me. I feel my role in life has changed a lot since the beginning of trying. You know, you feel less of a woman. I don’t feel that role is fulfilled and it’s just a missing spot in myself.” (11)	1–7, 9–23, 25, 26, 29, 30, 32–34, 36–38, 40–45, 47
	Loss of agency		“Helplessness, frustration, a little anxiety, stress …, you don’t really know what’s happening to you … really helpless, really helpless …” (15)	1, 2, 6, 7, 9–13, 15–22, 24, 26–29, 31, 32, 35–37, 40, 41, 43–48
Connection to others	Place in community is uprooted	Painful questions and jokes	“I feel uncomfortable meeting with people who know that I am infertile because they bombard me with questions just to satisfy their curiosity. I do not want to talk to them. It is not appropriate to tell them ‘do not ask me questions like these’ so I do not talk to them at all. I, even, distance myself from my co-workers for this reason.” (23)	1–14, 16, 18, 20–27, 29–32, 34–38, 40, 41, 43–48
		Exclusion from social gatherings	“If any functions like baby shower and baby birthday happen in their house. They ignore us by not inviting us to that function and not informing us about the functions at all.” (44)	3 -5, 14, 21, 22, 36, 44
		Feeling misunderstood	Barbara (…) had entrusted a close friend with information about her fertility problem, yet described feeling hurt and betrayed when her friend asked Barbara if she was pregnant yet. Barbara explained, “I was just like, I was flabbergasted. I thought, how am I in this situation right now? It was a terrible feeling…She should’ve known not to ask me.”(8)	1–8, 10, 11, 13, 14, 16–18, 20, 21, 23–32, 34, 36–38, 40, 43–48
		Nonjudgmental support	“My husband has always supported me. When I have a lot of problems, I also share them with my mother and sisters. I have always received support from them.” (36)	4, 8, 9, 14, 17–20, 23, 27–29, 32–34, 36, 40, 42, 43, 45
	Searching for belonging		“... Is it not awful really?... That those I actually know engage less with me, my husband and what we are going through than you do on this account. On the other hand, that makes me all the more humble and grateful to have this account, and above all YOU.” (Instagram post, March 2019) (47)	2, 4, 6, 9, 15, 18, 19, 28, 29, 31, 32, 35, 37, 45–47
	Impact on the relationship	Partners pull together	‘‘When we started the process we were both ready and I believe it [the experience] has made our relationship stronger.’’ (man) (35)	3, 4, 7, 9–11, 14, 15, 17, 18, 20–25, 30–33, 35–37, 40, 41, 43, 45, 48
		Pressure on the relationship	“My tests are clear and the issue is with her tubes. I understand her thoughts of "it’s her body, her issues, her grief" but it’s driving us apart. What is my role? Do I just keep quiet or how can help her see I’m there to support and take an active role?”(37)	1, 3, 4, 5, 7, 12–14, 16, 17, 20–25, 30–32, 34, 37, 38, 40, 41, 43–45, 48
Search for meaning and purpose	Trying to make sense of the situation		‘‘Because of the failed attempts through the IUIs, by the time we got to IVF, I just felt that maybe it wasn’t meant to happen … I started thinking, ‘maybe my body isn’t meant to have a child.’’’ (27)	2, 6, 7, 9–13, 17–20, 22, 24–33, 35–37, 40–48
	Relating to faith and religion	Faith as an anchor in life	“Religiously, it affirmed my faith in God in that I believed that I have a God who is able to do all things and that if you have God, you have every thing.” (26)	3, 4, 7, 22–24, 26–30, 32, 33, 36, 38, 42, 44, 48
		Faith as an obstacle	“I don’t know how to put it, whether it is God or nature, you sometimes question God, and think, “He is unjust.” [But then,] you ask, “Is there a God?” because I see that He grants kids to those who throw them on the street, who do not want them, and who commit crimes. What could I have done to deserve such punishment? What crimes did I do to serve this sentence.” (25)	3–5, 23–25, 28, 29, 32, 42, 44, 48
	Active reorientation		“We have taken care of our niece every weekend since she was born. She has brought us many happy times and makes us feel that we are parenting. We plan to leave all our property to her when we die.” (30)	4, 6, 12, 20, 28, 30, 32, 35, 38, 40
